# The nurse's role in managing gout in the modern era: A systematic review of the literature

**DOI:** 10.3892/mi.2023.100

**Published:** 2023-08-07

**Authors:** Paraskevi Tsiamalou, Alexandros G. Brotis, Eleni Vrekou, Vasiliki Epameinondas Georgakopoulou, Petros Papalexis, Aikaterini Aravanatinou-Fatorou, Maria Tegousi, George Fotakopoulos, Konstantinos Paterakis

**Affiliations:** 1 Department of Rheumatology, General University Hospital of Larissa, 41221 Larissa, Greece; 2 Department of Neurosurgery, General University Hospital of Larissa, 41221 Larissa, Greece; 3 Department of Infectious Diseases and COVID-19 Unit, Laiko General Hospital, Medical School, National and Kapodistrian University of Athens, 11527 Athens, Greece; 4 Unit of Endocrinology, First Department of Internal Medicine, Laiko General Hospital, Medical School, National and Kapodistrian University of Athens, 11527 Athens, Greece; 5 Department of Biomedical Sciences, University of West Attica, 12243 Athens, Greece; 6 First Department of Internal Medicine, Laiko General Hospital, Medical School, National and Kapodistrian University of Athens, 11527 Athens, Greece

**Keywords:** nurse, role, gout, uric acid arthritis, urate-lowering therapy, education

## Abstract

The current treatment of gout is largely suboptimal, with up to 89% of hospitalizations being preventable due to inadequate care. The present study performed a systematic review in an aim to identify barriers to optimal gout treatment (Q1), understand how frequently nurses are involved in the management of gout (Q2), and examine the role of the nurse in the management of gout (Q3). A systematic review was performed, focusing on studies reporting on the nurse's role in the management of gout and the quality of the gathered items was appraised based on the risk of bias. In total, 15 records fulfilled the eligibility criteria and were used in the present systematic review. The main barriers were attributed to the patient's experiences with gout and lay beliefs, which affected seeking advice and adherence to treatment (Q1). Recently, however, several advances in patient care, including nurse-led clinics, have expanded the nurse's role, accounting for as much as 26% of the annual visits (Q2). Nurse-led interventions, such as education and lifestyle counseling, increased adherence to treatment (Q3). On the whole, nurses are key players in multidisciplinary teams and should be capable of engaging in shared decision-making processes, goal setting, providing patients with education and information, and making appropriate referrals.

## Introduction

Gout flares are treated using anti-inflammatory agents, such as corticosteroids, non-steroidal anti-inflammatory drugs and colchicine, while allopurinol is the first-line drug used in urate-lowering therapy (ULT) to dissolve urate crystals, suppress gout flares and resolve tophi (1,2). However, the current treatment strategies are largely suboptimal, with up to 89% of hospitalizations being preventable owing to inadequate care (3). Recently, the nurse's role in managing gout has expanded (4) to include members of multidisciplinary teams (2), whereas the number of nurse-led clinics has increased with promising results (5,6). The present study aimed to identify barriers to optimal gout treatment (Q1), understand how frequently nurses are involved in the management of gout (Q2), and examine the role of the nurse in the management of gout (Q3).

## Data and methods

### Sources and study selection

A systematic review was performed, focusing on published evidence. The computerized literature search involved three databases (PubMed, Google Scholar and Scopus). The search criteria used are depicted in Table I. Two authors (VEG and PT) assessed the titles and abstracts of studies to eliminate records based on the study design. Additional studies were discarded after reading the full-text document. The reference lists of the gathered records were searched for additional citations. Two authors (MT and PT) appraised the quality of the gathered items based on the risk of bias, independently. Consensus and randomized controlled studies were considered to have a ‘low risk’ of bias, observational studies had ‘intermediate risk’, and case series and qualitative studies had a ‘high risk’. The overall quality of the available evidence was assessed using the GRADE recommendations (7).

## Results

### Literature search results

**Figure 1:**
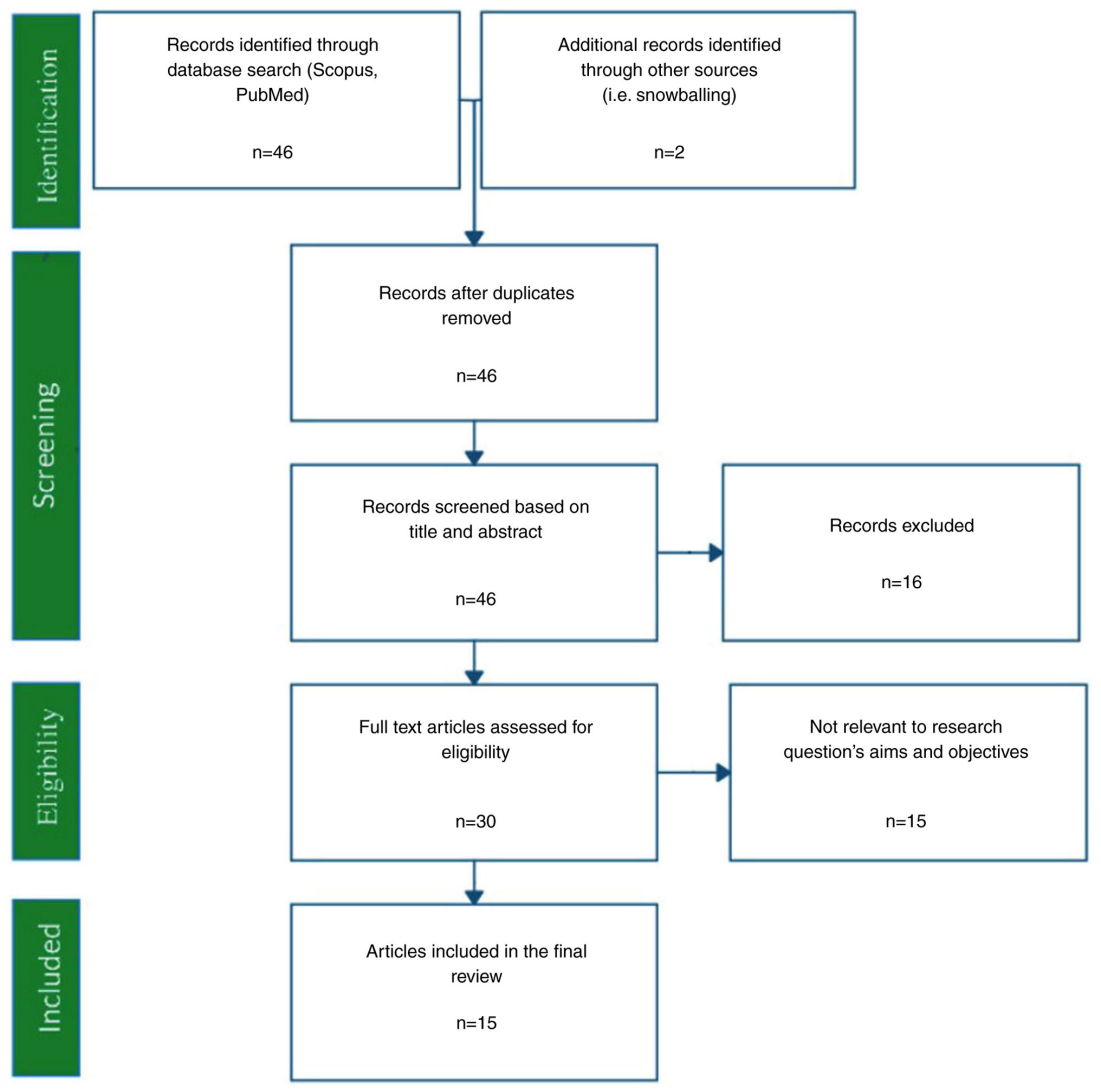
PRISMA flow chart of the systematic literature review.

**Figure 2:**
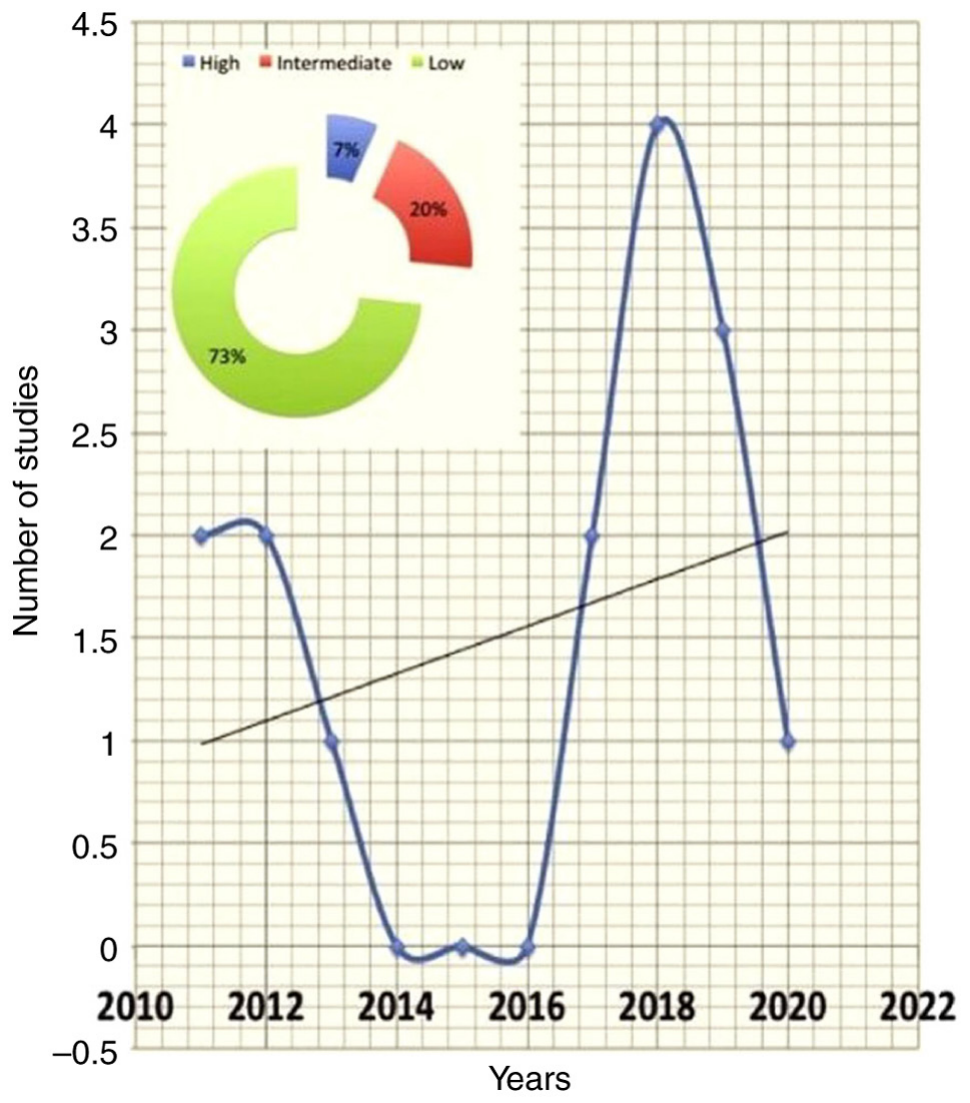
Number of included studies per year and percentages of low, intermediate and high quality.

**Figure 3:**
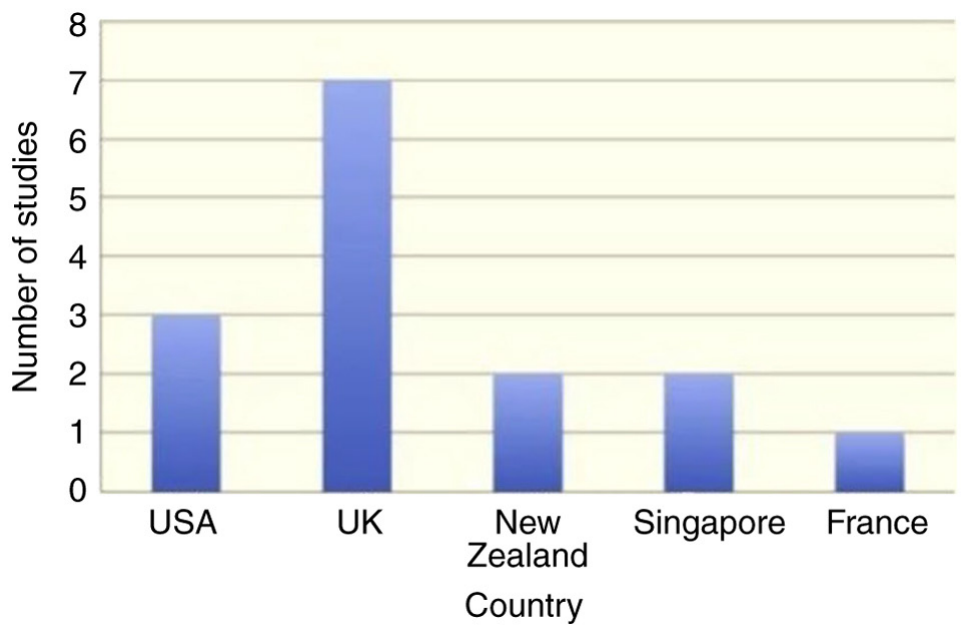
Number of included studies per country.

**Figure 4:**
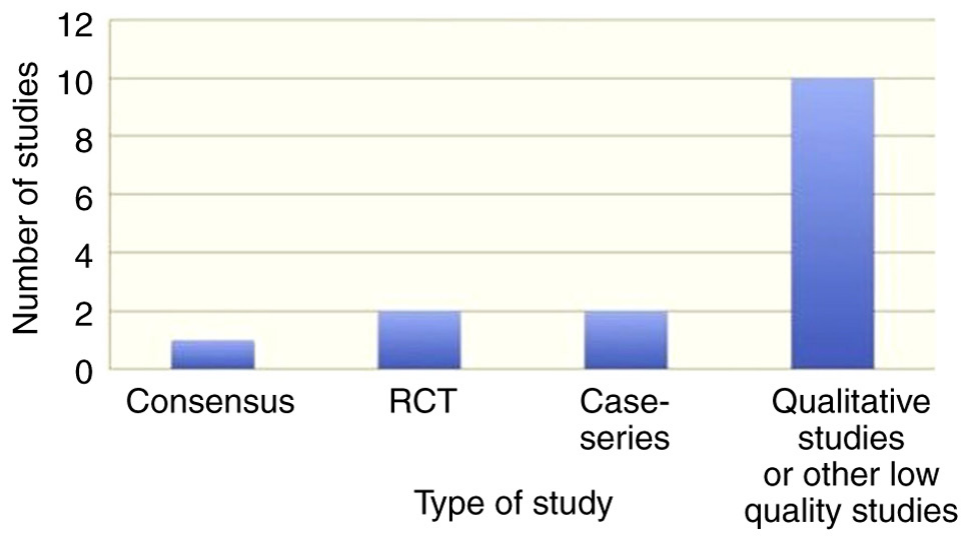
Types of included studies.

The literature search resulted in 46 records, and two additional studies were traced through the references (Fig. 1). In total, 15 records fulfilled the eligibility criteria and were used in the present systematic review (Tables II and III). The gathered items included one consensus, two randomized controlled trials, one review, one longitudinal study, two cross-sectional studies, three case series and five qualitative studies. The majority of the studies were conducted in the UK and the USA. The ‘high’, ‘intermediate’ and ‘low’ quality studies accounted for 7, 20 and 73% of the available evidence, respectively (Fig. 2, Fig. 3 and Fig. 4). The data obtained on the role of on nurses' role in managing gout were categorized into the following:

### Barriers to optimal gout treatment (Q1)

There is a small body of very low-quality evidence regarding Q1 (Tables IV and V). The main barriers were attributed to the patients' experiences with gout and lay beliefs, which affected seeking advice and adherence to treatment (8,9). Misconceptions were preserved by the lack of understanding of the causes and consequences of the disease and its response to lifestyle change and the use of ULT (8,9). Gout was considered self-inflicted or part of aging (8). The majority focused on managing acute attacks rather than treating the underlying cause (8). The lack of knowledge by the health professionals was reflected in the suboptimal information given to patients and the reluctance to offer ULT as a ‘curative’ long-term management strategy (8). The gaps in knowledge were attributed to the absence of formal education on the topic. Spouses highlighted that general practitioners did not have time to educate patients (9). During the flare of gout, feelings of powerlessness led to a delay in seeking medical attention and patient withdrawal (9). Nurses regretted that they did not have a sufficient amount of time to discuss issues with patients (9). Several patients raised concerns regarding the long-term use of ULT related to prescription costs, polypharmacy, relative contraindications, overuse, dependency, side-effects and long-term effects on health (10). Hearsay about these side effects prevented them from following their doctor's prescriptions (11). The female sex, marriage, the absence of distracting factors (job) and the presence of multiple co-morbidities were associated with higher rates of treatment adherence (12).

### Frequency with which nurses are involved in the management of gout (Q2)

There is limited evidence of poor quality with regard to Q2. Singh *et al* (13) conducted a survey of 298 patients with gout from three metropolitan areas to study healthcare utilization patterns. The most utilized gout-related health care resource was the primary care physicians, used by 60.4% of patients with a mean annual utilization of 3.1 (SD 3.4) visits (13). Visits to rheumatologists and nurse practitioners followed with 50.7% (3.7±5.7 annual visits) and 26% (2.7-2.5 annual visits), respectively (13). Nurse practitioners, physician assistants, urgent care and emergency department resources were each used by approximately 1/4 of patients, averaging ~2 visits per year (13). Overnight hospitalization for gout was reported by #x003C;10% of patients (13).

### Role of the nurse and its effectiveness (Q3)

The evidence regarding Q3 is sufficient and of high quality. A consensus for gouty arthritis agreed that ‘multidisciplinary referral provides optimal care in cases of recalcitrant gout’ and that ‘patient education should include dietary modification, medication adherence, and follow-up care with their assigned health care providers’ (4).

Indeed, increased adherence to treatment was achieved by nurse-led interventions, including education and lifestyle counseling (14,15). The educational programs provided detailed information about the cause of the disease and known risk factors, the risk of irreversible joint damage and treatment options, such as individualized risk factor modification and ULT (14). The success rate of these nurse-led interventions in achieving the therapeutic target (SUA ≤360 µmol) at 12 months was as high as 92% (14), which remained at the 5-year follow-up (16). Other programs included a nursing educational intervention via a structured curriculum and monthly follow-up calls from pharmacists to emphasize adherence to management programs) (17). The majority of subjects confirmed the usefulness of the overall program in understanding and managing their gout and appreciated the role of the nurses and pharmacists in 81 and 50% of the responders, respectively (17). Furthermore, these programs corrected misconceptions about bridge therapy, the possibility of being flare-free, and the genetic component of gout (17).

The Nottingham Gout Treatment Trial compared nurse-led gout care to usual care led by general practitioners for people in the community in a randomized controlled study (5). More patients receiving nurse-led care had serum urate concentrations #x003C;360 µmol/l at 2 years than those receiving usual care (risk ratio, 3.18; 95% confidence interval, 2.42-4.18) (5). Moreover, the cost per quality-adjusted life year gained for the nurse-led intervention was £5,066 at 2 years (5). Participants described that the nurse-led intervention facilitated engagement with ULT, namely by providing improved knowledge and understanding of gout and its treatment, involvement of patients in decision-making about treatment, increased confidence about the benefits of treatment, and encouragement to persist with ULT (10). Among the reasons for ULT discontinuation in the nurse-led arm were the absence of flares, experience of side-effects, being frustrated with taking the tablets, and the interruption of ULT prescription by the general practitioner (6).

McLachlan *et al* (18) investigated a nurse-led multidisciplinary approach to improving CVD risk management in patients with gout. Any areas the patient with a 5-year CVD risk >10% felt willing and confident to manage (smoking cessation, physical activity, healthy eating, adherence to medication) were addressed through self-management support and encouragement (18). The prescription of aspirin, statins, nicotine replacement therapy, uptake of self-reported activity levels, and mean systolic and diastolic blood pressure, markedly improved after 6 months (18).

Finally, it has been recognized that community-based nurses require competencies to enable them to assess, care for and manage arthritis appropriately (19). These competencies included an understanding of the underlying pathology, the ability to distinguish between the various types, and the ability to recognize early warning signs, with an emphasis on osteoarthritis, rheumatoid arthritis, gout and septic arthritis (19). In addition, nurses should be capable of engaging in shared decision-making processes, goal setting, providing patients with education and information, and making appropriate referrals (19). In the Nottingham Gout Treatment Trial, the nurses received specialized training in the management of gout, including providing individualized information and engaging patients in shared decision-making (5).

## Discussion

**Figure 5:**
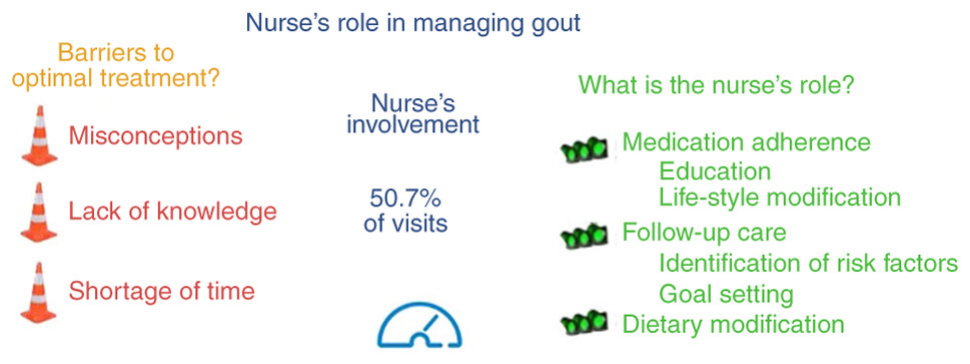
Summary of the current findings.

It has become apparent that the absence of proper information prevents patients from adhering to optimal gout treatment. Since nurses are involved in all phases of gout treatment and are trusted in up to a fourth of the cases, the specialized ‘rheumatology nurse’ concept has gained acceptance in a limited number of countries, such as the UK and USA. Rheumatology nurses have been adequately educated on all theoretical aspects of gout, deliver a structured curriculum to the patients, and have been trained in goal setting and engaging the patient in the individualized shared-decision-making process. As a result, institutions utilizing the ‘specialized rheumatology nurse’ exhibited higher adherence rates to ULT, an increased control of gout flares and an improved quality of life of patients with gouty (Fig. 5).

The concept of a ‘specialized nurse’ is not novel. Pediatric nurse practitioners have been effective in promoting breastfeeding (20). Nurse-led clinics have also been very successful in managing hypertension and diabetes mellitus (21,22). Emphasis should be placed on the concept of multidisciplinary teams, where each member plays a discrete role and works in harmony with the others. Frequently, a rheumatologist constructs the curriculum and educates the other members of the team (17). The nurses then deliver the lessons to the patients, tailor the management to the patient's needs and preferences, and monitor adherence to treatment goals and outcomes. In Japan, it is common for patients to receive dietary guidance from a general physician after abnormal values are found during a physical examination (23). Enlightenment is provided through television health programs (24). The training of nurses with specialized knowledge is taking place, although in small numbers (25). Patient education that provides detailed guidance to a wide range of patients is more effective by nurses (25). In some programs, pharmacists are also involved in patient motivation (17).

The present systematic review has some critical limitations which should be mentioned. It was based on a limited number of studies, of which the majority are qualitative in design with a significant risk of bias. However, it should be noted that among the included studies, there were two well-conducted randomized controlled studies (5,6) and the results of a consensus meeting (4). The majority of the studies were conducted at a single center (5,6,10,14,16), thus questioning the generalizability of the findings. Further studies are thus required to validate the efficacy and efficiency of the multidisciplinary teams in more high-quality studies, considering the individual characteristics of various countries and populations.

In conclusion, the present study demonstrated an expanded role of nurses regarding the management of gout. Thus, nurses are invited to improve their understanding of gout, its pathogenesis and treatment, and to be capable of goal-setting and shared decision-making. The goal is to educate patients with gout to adhere to ULT and improve their quality of life without increasing costs.

## Figures and Tables

**Table I:** Search eligibility criteria of the systematic review according to PICO.

Frame	P (patients, participants, population)	I (intervention)	C (comparator/ reference test)	O (outcome)	Time	
Mesh terms	#1. Adults #2. ‘Gout’ OR ‘Uric acid arthritis’ #3. ‘Nurse’ OR ‘Nursing’ #4. English language	#5. ‘Any’	#6. ‘Any’	#7. ‘Any’	Search period duration: 2010 to 2020	Last search: April, 2020
Search	#1 AND #2 AND #3 AND #4 AND #5 AND #6 AND #7					
Example in Scopus:	[TITLE (nurse) OR TITLE (nursing) AND TITLE-ABS-KEY (gout) OR TITLE-ABS-KEY (uric AND acid AND arthritis)] AND PUBYEAR >2010					
Exclusion criteria	Irrelevant title or abstract, irrelevant full-text, editorial, reviews, case-reports, meta-analysis, pediatric/neonatal studies, experimental/nonhuman studies, non-English studies, experimental studies.					
Sources	Databases (PubMed and Scopus)					
	Reference list					

**Table II:** Bibliometric characteristics of the eligible studies.

Author(s)	Year	Country	Citations	Level of Evidence	Journal	Impact factor of Journal	Study design	Question	(Refs.)
MacLachan *et al*	2011	New Zealand	6	4	Eur J Cardiovasc Nurs	2.65	Longitudinal study	Q3	(18)
Singh *et al*	2011	USA	34	5	Semin Arthritis Rheum	5.07	Cross-sectional study	Q2	(13)
Spencer *et al*	2012	UK (Nottingham)	116	5	Annals of the Rheumatic Diseases	14.30	Qualitative	Q1	(8)
Dalbeth	2012	New Zealand	0	5	Nature Reviews Rheumatology	18.54	Review	Q3	(15)
Rees *et al*	2013	UK (Nottingham)	171	4	Annals of the Rheumatic Diseases	14,30	Prospective case series	Q3	(14)
Fields *et al*	2017	USA	17	5	Semin Arthritis Rheum	5.07	Case series	Q3	(17)
Abhishek *et al*	2017	UK (Nottingham)	22	5	Rheumatology	5.25	Cross-sectional study	Q3	(16)
Seow *et al*	2018	Singapore	2	5	Clinical Nursing Research	1.50	Qualitative	Q1	(11)
Chua *et al*	2018	Singapore	4	5	Journal of Clinical Nursing	1.76	Case series	Q1	(12)
Doherty *et al*	2018	UK (Nottingham)	27	1b	The Lancet	59.10	Randomized controlled trial	Q3	(5)
Erwin *et al*	2018	UK (Cardiff)	4	5	Musculoskeletal Care	(-)	Qualitative	Q3	(19)
Mirmarin *et al*	2019	USA	1	1a	The Journal of Foot and Ankle Surgery	1.04	Consensus	Q3	(4)
Latif *et al*	2019	UK (Nottingham)	4	5	Joint Bone Spine	3.28	Qualitative	Q3	(10)
Deprouw *et al*	2019	France	0	5	Joint Bone Spine	3.28	Qualitative	Q1	(9)
Fuller *et al*	2020	UK (Nottingham)	5	1b	Rheumatology (Oxford)	7.04	Randomized controlled trial	Q3	(6)

**Table III:** Summary table with the basic characteristics of the eligible studies.

Author(s)	Year	Participants	Intervention	Comparator	Outcome	Timing	(Refs.)
MacLachan *et al*	2011	Patients with gout and initial 5-year risk N10% for cardiovascular disease	Nurse-led multidisciplinary		The prescription of aspirin, statins, nicotine replacement therapy, uptake of self-reported activity levels, mean systolic and diastolic blood pressure, with a trend towards reduced cigarette smoking		(18)
Singh *et al*	2011	Patients with gout	(-)	(-)	Gout-related health care utilization over	1 year	(13)
Spencer *et al*	2012	Mixed patients with gout and care-givers	(-)	(-)	Patient and health care providers beliefs and experiences about causes and consequences of gout	(-)	(8)
Dalbeth	2012	Patients with gout	Package of care	(-)	(-)	(-)	(15)
Rees *et al*	2013	Patients with gout	Nurse-delivered intervention that included education, individualized lifestyle advice and appropriate ULT.	(-)	Percentage of patients who had their SUA reduced below 360 µmol/l at	1 year	(14)
Fields *et al*	2017	Patients with gout	Multi-disciplinary team gout education and management program	(-)	Subject and provider program evaluation questionnaires at 6 and 12 months, program retention rate and success in reaching patients via monthly calls.	6 and 12 months	(17)
Abhishek *et al*	2017	Patients with gout	Nurse-delivered intervention that included education, individualized lifestyle advice and appropriate ULT.	(-)	Persistence and adherence on ULT	5 years	(16)
Seow *et al*	2018	Patients with gout	(-)	(-)	Living with gout	(-)	(11)
Chua *et al*	2018	Patients with gout	(-)	(-)	Medication adherence, gout perception, relations with doctors, ULT, social support	(-)	(12)
Doherty *et al*	2018	Patients with gout	Nurse-led care	General practitioner- led care	Percentage of participants who achieved serum urate concentrations less than 360 µmol/l (6 mg/d), flare frequency, presence of tophi, quality of life, and cost per quality-adjusted life-year (QALY) gained.	2 years	(5)
Erwin *et al*	2018	Expert panel	(-)	(-)	Competencies needed by community- based nurses and AHPs	(-)	(19)
Mirmarin *et al*	2019	American College of Foot and Ankle Surgeon and the American Association of Nurse Practitioners	(-)	(-)	(-)	(-)	(4)
Latif *et al*	2019	Patients with gout	Nurse- led complex package	(-)	Perception of the role of the nurse in engagement with the ULT	18-26 months after	(10)
Deprouw *et al*	2019	Partners and nurses	(-)	(-)	Knowledge and representations		(9)
Fuller *et al*	2020	Patients with gout	Nurse-led care	General practitioner- led care	Satisfaction, knowledge, flairs	Over 1 year	(6)

AHPs, allied health professionals; SUA, serum uric acid; ULT, urate lowering treatment; QALY, quality-adjusted life-year.

**Table IV:** Grading of the retrieved articles in regard to the Quality of Evidence.

		Down-grade					Up-grade			
Question	Starting grade	Risk of bias	Inconsistency	Indirectness	Imprecision	Publication bias	Magnitude of effect	Dose response	Confounding factors	Final grade
What are the barriers for the optimal gout treatment? (Q1)	2	-1	0	0	0	-1	+1	0	0	1
How frequently are nurses involved in the management of gout (Q2)	1	0	0	0	0	-1	0	0	0	0
What is the role of the nurse and is it effective? (Q3)	4	0	0	0	0	0	+1	0	0	5

**Table V:** Summary-of-evidence table.

Question	Studies	Citations	Quality of pertinent evidence (GRADE)	Relative risk (95% CI)	Conclusions	Future recommendations
What are the barriers for the optimal gout treatment? (Q1)	4	122	Very low quality	(-)	There is a small body of very low-quality evidence that the main barriers for the optimal gout treatment are related to the lack of patient education on gout causes, risk factors, treatment modalities, including the ‘curative’ long-term management strategy	Further evidence from high=quality studies is needed to validate the evidence across countries and populations
How frequently are nurses involved in the manage ment of gout (Q2)	1	34	Very low quality	(-)	There is a small body of very low-quality evidence that the nurse practitioners are used by about 1/4 of patients, averaging approximately 2 visits per year	High=quality studies are needed to quantify the frequency of nurse involvement across different countries and populations
What is the role of the nurse and is it effective? (Q3)	10	253	High quality	3.18 (2.42-4.18)	More patients receiving nurse-led care had serum urate concentrations less than 360 µmol/l at 2 years than those receiving usual care. Meanwhile, the cost per QALY gained for the nurse-led intervention was £5,066 at 2 years	High=quality studies are required to identify the role of the clinical nurse in physician-led clinics

## Data Availability

The datasets used and/or analyzed during the current study are available from the corresponding author on reasonable request.
